# Ultra-treatment-resistant Schizophrenia. A case report

**DOI:** 10.1192/j.eurpsy.2022.2019

**Published:** 2022-09-01

**Authors:** A. Osca Oliver, M. Palomo Monge, M. Pérez Fominaya, M.V. López Rodrigo, M.F. Tascón Guerra

**Affiliations:** 1 Hospital Nuestra Señora del Prado, Psiquiatría, Talavera de la Reina, Spain; 2 Hospital Nuestra Señora del Prado, Psiquiatria, Talavera de la Reina, Spain

**Keywords:** schizophrénia, treatment resistant, refractory schizophrenia, Antipsychotics

## Abstract

**Introduction:**

Despite the efficacy of antipsychotics, up to about 30% of schizophrenia patients do not respond adequately to treatment and are called treatment-resistant schizophrenia (TRS) patients. The treatment of choice in these patients is clozapine, which is used last due to the adverse effects it can cause. However, it has been shown that half of TRSs are also resistant to clozapine, leading to ultra-resistant schizophrenia.

**Objectives:**

We present a clinical case corresponding to a 33-year-old man, single, residing in a community residence, undergoing psychiatric follow-up from the age of 7, receiving during this period the diagnoses of schizotypal personality disorder and paranoid schizophrenia.

**Methods:**

As of 2015, he began to make autolytic attempts, the last being this year, 2021. Moment in which he manifests for the first time presenting imperative, sporadic auditory pseudo-hallucinations, which incite self-harm. These sensory-perceptual alterations appeared from 2015, together with the worsening of the negative symptoms.

**Results:**

The patient has been treated with numerous antipsychotics, without complete remission, so since 2019 treatment with Clozapine 200mg was started. As the symptoms did not subside, the dose was increased to 400mg, at which point some of its side effects began to appear; urinary incontinence, sedation, sexual impotence … so the patient abandoned the treatment, suffering a relapse of his mental pathology.

**Conclusions:**

Despite the arrival of atypical antipsychotics, it remains a challenge that there is a complete remission of symptoms in some patients with schizophrenia, for which we consider that psychopharmacological research in this group of patients is of the utmost importance.

**Disclosure:**

No significant relationships.

